# Supplementary material for: Public perceptions of physician-pharmaceutical industry relationships and trust in physicians
Source: PLoS One. 2023 Nov 28;18(11):e0294854. doi: 10.1371/journal.pone.0294854 (PMC10684105; doi:10.1371/journal.pone.0294854)
Supplement: S1 File — (DOCX) [file pone.0294854.s001.docx]

**S1. The survey instrument in English**

If you agree to participate in the survey, please check (✔) the box and begin answering.

□ I agree.

1. For each of the following, please select one option that applies.
2. Have you ever noticed when visiting medical facilities, that physicians use office goods (e.g., pens or notepads) with the name of a pharmaceutical company or drug on them?
3. Yes
4. No
5. Have you ever noticed that promotional materials with the name of a pharmaceutical company or drug (e.g., calendars or organ models) are placed in the examination rooms or waiting areas of medical facilities?
6. Yes
7. No
8. Have you ever noticed a representative of a pharmaceutical company present in the examination rooms or waiting areas of medical facilities?
9. Yes
10. No
11. For each of the following, please select one option that best represents your personal acceptance of the situation.
12. Physicians receiving pens with the name of a pharmaceutical company or drug from the company.
13. Acceptable
14. Somewhat Acceptable
15. Neutral
16. Somewhat unacceptable
17. Unacceptable
18. Physicians receiving pens with the name of a pharmaceutical company or drug from the company.
19. Acceptable
20. Somewhat Acceptable
21. Neutral
22. Somewhat unacceptable
23. Unacceptable
24. Physicians attending promotional drug seminars (where the company provides information about the features, effectiveness, and side effects of its drugs) within medical facilities.
25. Acceptable
26. Somewhat Acceptable
27. Neutral
28. Somewhat unacceptable
29. Unacceptable

1. Physicians receiving meal offerings from pharmaceutical companies at restaurants.
2. Acceptable
3. Somewhat Acceptable
4. Neutral
5. Somewhat unacceptable
6. Unacceptable
7. For each of the following, please select one option that applies.
8. Do you think physicians’ receiving information about a company's drug from a pharmaceutical company representative influences their prescription habits?
9. Very influential
10. Influential
11. Neutral
12. Not very influential
13. Not influential
14. Do you think physicians’ receiving pens with the name of a company or drug from a pharmaceutical company influences their prescribing habits?
15. Very influential
16. Influential
17. Neutral
18. Not very influential
19. Not influential
20. Do you think physicians’ receiving meal offerings from pharmaceutical company representatives at restaurants influences their prescribing habits?
21. Very influential
22. Influential
23. Neutral
24. Not very influential
25. Not influential

4. For each of the following, please select one option that applies.

1. What proportion of physicians do you think receive office goods (e.g., pens) from pharmaceutical companies?
2. Almost all physicians are receiving them
3. Many physicians are receiving them
4. About half of the physicians are receiving them
5. A small number of physicians are receiving them
6. Most physicians are not receiving them
7. What proportion of physicians do you think receive meal offerings at restaurants from pharmaceutical companies?
8. Almost all physicians are receiving them
9. Many physicians are receiving them
10. About half of the physicians are receiving them
11. A small number of physicians are receiving them
12. Most physicians are not receiving them
13. Would you like to know about the relationship your primary care physician has with pharmaceutical companies? (If you don't have a primary care physician, assume it's the physician who sees you.)
14. Would like to know
15. Somewhat interested
16. Neither
17. Would prefer not to know
18. Would not like to know
19. For each of the following, please select the one option that applies the most.
20. Physicians in general care about their patients' health just as much or more as their patients do.
21. Strongly agree
22. Agree
23. Neutral
24. Disagree
25. Strongly disagree
26. Sometimes physicians care more about what is convenient for them than about their patients' medical needs.
27. Strongly agree
28. Agree
29. Neutral
30. Disagree
31. Strongly disagree
32. Physicians are extremely thorough and careful.
33. Strongly agree
34. Agree
35. Neutral
36. Disagree
37. Strongly disagree
38. You completely trust physicians' decisions about which medical treatments are best.
39. Strongly agree
40. Agree
41. Neutral
42. Disagree
43. Strongly disagree
44. Physicians are totally honest in telling their patients about all of the different treatment options available for their conditions.
45. Strongly agree
46. Agree
47. Neutral
48. Disagree
49. Strongly disagree
50. Physicians think only about what is best for their patients.
51. Strongly agree
52. Agree
53. Neutral
54. Disagree
55. Strongly disagree
56. Sometimes physicians do not pay full attention to what patients are trying to tell them.
57. Strongly agree
58. Agree
59. Neutral
60. Disagree
61. Strongly disagree
62. Physicians always use their very best skill and effort on behalf of their patients.
63. Strongly agree
64. Agree
65. Neutral
66. Disagree
67. Strongly disagree
68. You have no worries about putting your life in the hands of physicians.
69. Strongly agree
70. Agree
71. Neutral
72. Disagree
73. Strongly disagree
74. A physician would never mislead you about anything.
75. Strongly agree
76. Agree
77. Neutral
78. Disagree
79. Strongly disagree
80. All in all, you trust doctors completely.
81. Strongly agree
82. Agree
83. Neutral
84. Disagree
85. Strongly disagree

7. We would like to ask you about yourself.

1) Gender (Please choose one)

1. Male

2. Female

3. Other

1. Age (Please fill in the number)

[ years old]

1. Do you have a primary care physician (or family doctor)?
2. Yes
3. No
4. Do you have a primary care physician (or family doctor)?
5. Yes
6. No
7. What is your current health status? Please select the one that applies the most.
8. Very good
9. Good
10. Average
11. Not so good
12. Very poor

This concludes the survey. Thank you for your valuable time.
