# Supplementary material for: Public perceptions of physician-pharmaceutical industry relationships and trust in physicians
Source: PLoS One. 2023 Nov 28;18(11):e0294854. doi: 10.1371/journal.pone.0294854 (PMC10684105; doi:10.1371/journal.pone.0294854)
Supplement: Attachment — Submitted filename: Response to Reviewers.docx [file pone.0294854.s003.docx]

Dear Dr. Soham Bandyopadhyay,

We are submitting a revised version of our manuscript titled “Public perceptions of physicians-pharmaceutical industry relationships and trust in physicians” (reference number PONE-D-23-18061) for consideration for publication in PLOS ONE.

We are grateful for the constructive comments provided by the reviewer. These suggestions have been instrumental in enhancing the quality of our manuscript. We have addressed each comment in detail. All revisions have been highlighted in yellow, and any deletions are indicated with a strikethrough. We have also inserted our comments in the manuscript addressing both the corrections and comments made by the reviewer.

We hope that our revised manuscript meets the publication standards of PLOS ONE.

Sincerely,

Kei Mukohara, MD

Reviewer #1: Comparisons of the results with previous studies from the U.S and Turkey for example, could be made clearer and less complicated.

Response: Thank you for your feedback regarding the clarity of comparisons between our results and previous studies from the U.S. and Turkey. Based on your feedback, we revised the sentences as follows: " In our study, fewer participants were aware of office stationery in examination rooms and waiting areas compared to prior U.S. studies [8,18,19]. However, their awareness of promotional items such as calendars and the presence of PRs was consistent with a previous study [8]. Regarding the acceptability of gifts to physicians, our participants' views on receiving stationery, medical textbooks, and meals at restaurants were in line with earlier findings [9,11,18,20]. They believed that stationery gifts had less influence on physicians than perceived by U.S. patients [11], but more than the views of patients in Turkey [21]. Additionally, our participants felt that gifts of meals influenced physicians more than the beliefs held by both U.S. and Turkish patients [11,21] "

Reviewer #1: In addition, the implications of the study towards physicians' prescription habit could be further fleshed out, as the paper only hints out on the role played by the Japanese culture and its healthcare system.

Response: We appreciate your insights. Our study primarily aimed to compare public perceptions with those of physicians on physician-industry relationships. We mentioned the Japanese culture and healthcare system in the limitations section to highlight potential constraints in the generalizability of our findings and not to suggest direct influences on physicians' prescribing habits.
